# OCP3 is an important modulator of NPR1-mediated jasmonic acid-dependent induced defenses in *Arabidopsis*

**DOI:** 10.1186/1471-2229-10-199

**Published:** 2010-09-13

**Authors:** Vicente Ramírez, Sjoerd Van der Ent, Javier García-Andrade, Alberto Coego, Corné MJ Pieterse, Pablo Vera

**Affiliations:** 1 Instituto de Biología Molecular y Celular de Plantas (IBMCP), Universidad Politécnica de Valencia-Consejo Superior de Investigaciones Científicas (CSIC). Camino de Vera s/n, Valencia, Spain; 2 Plant-Microbe Interactions, Department of Biology, Faculty of Science, Utrecht University, 3508 TB Utrecht, The Netherlands

## Abstract

**Background:**

Upon appropriate stimulation, plants increase their level of resistance against future pathogen attack. This phenomenon, known as induced resistance, presents an adaptive advantage due to its reduced fitness costs and its systemic and broad-spectrum nature. In *Arabidopsis*, different types of induced resistance have been defined based on the signaling pathways involved, particularly those dependent on salicylic acid (SA) and/or jasmonic acid (JA).

**Results:**

Here, we have assessed the implication of the transcriptional regulator OCP3 in SA- and JA-dependent induced defenses. Through a series of double mutant analyses, we conclude that SA-dependent defense signaling does not require OCP3. However, we found that *ocp3 *plants are impaired in a *Pseudomonas fluorescens *WCS417r-triggered induced systemic resistance (ISR) against both *Pseudomonas syrinagae *DC3000 and *Hyaloperonospora arabidopsidis*, and we show that this impairment is not due to a defect in JA-perception. Likewise, exogenous application of JA failed to induce defenses in *ocp3 *plants. In addition, we provide evidence showing that the over-expression of an engineered cytosolic isoform of the disease resistance regulator NPR1 restores the impaired JA-induced disease resistance in *ocp3 *plants.

**Conclusions:**

Our findings point to a model in which OCP3 may modulate the nucleocytosolic function of NPR1 in the regulation of JA-dependent induced defense responses.

## Background

To effectively combat invasion by a great variety of microbial pathogens, plants have evolved sophisticated strategies to monitor microbial populations and efficiently adapt to changes in their complex hostile environment. This responsive capacity is highly flexible and implicates a complex network of interactions between the different layers of the immune system. These include a first defensive barrier to hamper pathogen entry such as physical reinforcement of cell walls through production of callose and lignin (for a review see [1]). When this pre-invasive layer of defense is overcome, other defense systems are recruited to produce a battery of antimicrobial metabolites and proteins able to halt or dismiss pathogen invasion. The phytohormones salicylic acid (SA), jasmonic acid (JA), and ethylene (ET) have emerged as key players in regulating the activation of the basal defense responses involved in this second layer of the immune system (reviewed by [2-5]). The activation of plant defenses involves cross-talk between different hormonal pathways that finely tune the defense reaction depending on the nature of the intruder [6,7]. In general, pathogens with a biotrophic lifestyle are more sensitive to SA-dependent defense responses, whereas necrotrophic pathogens are primarily resisted by defenses dependent on JA, ET, or both [6-9]. Accordingly, *Arabidopsis *mutants that fail to produce, accumulate or perceive SA show enhance susceptibility to biotrophs. Likewise, mutations that disrupt JA signaling result in enhanced susceptibility to necrotrophic pathogens (reviewed by [9,10]).

In nature, plants often deal with simultaneous invasion by multiple aggressors, which can influence the primary induced defense response of the host plant [11-13]. There are many examples where an antagonism between SA and JA signaling pathways has been described [3,6,14,15]. In fact, accumulation of SA following pathogen infection strongly antagonizes JA-dependent defenses [16-18]. As a result of the negative interaction between SA and JA signaling, activation of the SA response should render a plant more susceptible to attackers that are resisted via JA-dependent defenses and *vice versa*. Indeed, many examples of trade-offs between SA-dependent resistance against biotrophic pathogens and JA-dependent defense against necrotrophic pathogens have been reported [12]. The JA-responsive *PDF1.2 *and *VSP2 *marker genes and several genes of the octadecanoid biosynthesis pathway have been identified as targets of the SA-mediated suppression of JA-responsive gene transcription [18,19]. This cross-talk mechanism may represent a flexible signaling network that allows the plant to respond more efficiently to the presence of pathogens [3,20,21]. However, in spite of its agronomic and evolutionary importance, the underlying molecular mechanisms of SA/JA cross-talk remains to a large extent still unknown.

In addition to basal resistance mechanisms that protect plants against virulent pathogens, plants have the ability to develop an enhanced defensive capacity against a broad spectrum of pathogens after stimulation by specific biological or chemical agents. In *Arabidopsis*, two forms of biologically induced disease resistance have been characterized: systemic acquired resistance (SAR), which is triggered upon infection by a necrotizing pathogen; and induced systemic resistance (ISR), which is triggered by colonization of roots by selected strains of non-pathogenic rhizobacteria [22-24]. SAR and ISR are both effective against different although overlapping subsets of pathogens, but they are regulated by distinct signaling pathways. SAR is characterized by an increase in SA levels, is associated with transcriptional activation of pathogenesis-related (*PR*) genes and is JA/ET-independent [25,26]. More recently, Truman et al. [27] and Attaran et al. [28] have presented additional evidences that represent opposing views on the role of JA signaling in modulating the establishment of SAR. Conversely, ISR functions independently of SA and requires components of the JA and ET signaling apparatus [26,29]. Despite the fact that the ISR mechanism is able to effectively protect several plant species (e.g. carnation, cucumber, radish, tobacco or *Arabidopsis*) against a wide range of pathogens, little is known about its molecular basis [30]. In *Arabidopsis*, analysis of local and systemic levels of JA and ET revealed that ISR is not associated with changes in the production of these two hormones and neither with major changes in transcript or protein profiles [29]. This suggests that ISR is based on the activation of yet unknown defense products. In any case, ISR establishment seems to involve the enhancement in the sensitivity to JA and ET rather than the increased production of any of these two hormones [31]. It has been hypothesized that the potentiation of plant defense responses involved in different types of induced resistance is mediated by an increase in the amount of latent cellular components with important roles in defense response signaling, a phenomenon called priming [30]. The increased presence of cellular signaling components might then lead to an accelerated and enhanced response when the cells are challenged by a second stress stimulus. Recently, evidence is accumulating that specific transcription factors, MAP kinases and secondary metabolites play an important role in the primed state of a plant [32-35].

NPR1 is a defense regulatory protein that was identified in *Arabidopsis *through several genetic screens for SAR-compromised mutants (reviewed by [14]). Subsequent studies revealed that NPR1 is a key regulator of induced resistances, including SAR and ISR (reviewed by [23,36]). During normal plant growth, the redox-sensitive NPR1 protein is present as an oligomer in the cytosol. Upon activation by SA, the redox state of the cytosol becomes more reduced, after which NPR1 is monomerized and translocated into the nucleus to function as a co-activator of the expression of *PR *genes [37]. Besides its crucial role in the regulation of SA-dependent defenses, which is predominantly exerted in the nucleus, an additional cytosolic function of NPR1 was identified in cross-talk between SA and JA signaling. In mutant *npr1-1 *plants, which do not produce a functional NPR1 protein, SA-mediated suppression of JA-responsive gene expression was shown to be abolished [18]). Using a dexamethasone-inducible system to control the nucleocytoplasmic localization of NPR1, it was demonstrated that a cytosolic function of NPR1 is crucial in this cross-talk process [18]. In addition, mutant *npr1-3 *plants, which produce a cytoplasmically-localized NPR1 protein lacking the C-terminal domain in which the nuclear localization signal is located, are only blocked in NPR1-dependent, SA-responsive gene expression while NPR1-dependent, JA/ET-regulated gene expression is relatively unaffected in this mutant [38]. Also SA-mediated suppression of JA/ET-responsive gene expression was shown to be unaffected in *npr1-3 *[39], corroborating the notion that the cytosolic function of NPR1 plays a role in the modulation of JA-dependent defenses [18,39-42].

Most studies have concentrated on unraveling the role of NPR1 in regulating SA-dependent SAR and *PR *gene expression (reviewed by [14]). However the involvement of NPR1 in the control of JA-dependent defenses is much less understood. Our current understanding suggests that nuclear NPR1 regulates SA-dependent gene expression and SAR establishment, whereas cytosolic NPR1 regulates SA-mediated suppression of JA-dependent defenses. Interestingly though, it has been demonstrated that it is possible to simultaneously activate SAR and ISR in *Arabidopsis*, and this results in an enhanced level of induced protection against *P. syringae *pv. *tomato *DC3000 (*Pst *DC3000). Furthermore, it indicates that these two induced resistance responses are compatible and additive [43]. Moreover, it suggests that plants can activate JA/ET-dependent defenses without negatively being affected by SA-dependent defenses.

Most of the mutants affected in the response to JA-mediated disease resistance against necrotrophs have opposite effects on SA-mediated disease resistance against biotrophs. This trade-off is generally explained by the antagonistic effect that exists between SA and JA signaling pathways [3,21,44]. Previously, we isolated and characterized the recessive *Arabidopsis ocp3-1 *mutant which shows enhanced disease resistance against the necrotrophic fungal pathogens *Botrytis cinerea *and *Plectosphaerella cucumerina*, but is not impaired in basal defense against the biotrophs *Hyaloperonospora arabidopsidis *and *Pst *DC3000 [45]. This phenotype is correlated with a constitutive activation of the JA-responsive *PDF1.2 *and the redox-sensitive *GST1 *marker genes. The enhanced disease resistance to necrotrophs of *ocp3-1 *mutant plants is fully dependent on COI1, a central regulator of JA-signaling [46-48]. The *OCP3 *gene encodes a homeodomain transcriptional factor which is constitutively expressed in healthy plants but repressed in response to infection by necrotrophic fungi and exogenous applications of MeJA and ABA [45,49]. In this work we further investigate the role of OCP3 in SA- and JA-mediated induced defenses. Our results reveal that OCP3 regulates specifically JA-dependent induced defenses, including *Pseudomonas fluorescens *WCS417r-triggered ISR and MeJA-induced disease resistance, but not basal defense. In addition we provide evidence pointing at a plausible mechanism by which OCP3 regulation of this process is based on the modulation of a cytosolic NPR1 function.

## Results

### SA-dependent defense signaling is not affected in *ocp3-1 *plants

**Figure 1:**
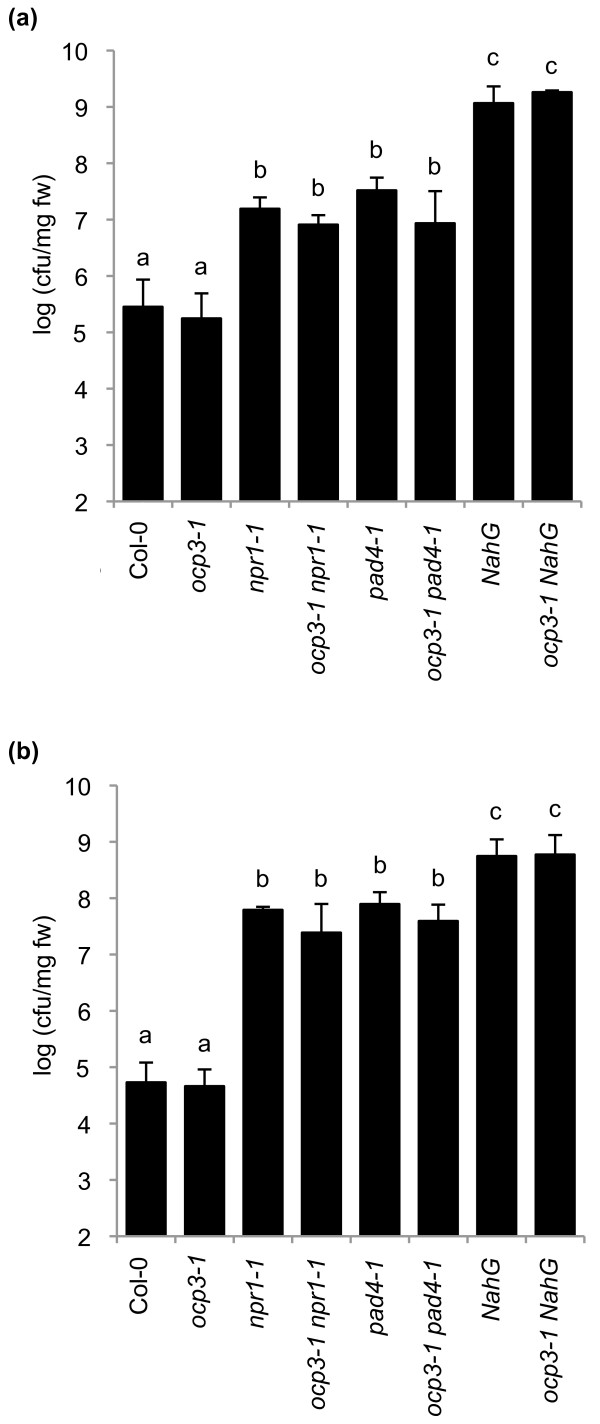
***OCP3 *is not implicated in the SA-dependent disease resistance signaling against *P. syringae***. Five-week-old plants were challenge inoculated with a bacterial suspension of virulent *Pst *DC3000 at the concentration of OD_600 _= 0.0004 (**a**) (Infiltration) or OD_600 _= 0.2 (**b**) (Spray). Three days after challenge inoculation, the bacterial growth was measured. Bars represent logarithmic units of colony forming units per cm^2 ^of leave. Error bars represent standard deviation (n = 16 plants). The experiments were repeated at least three times with similar results.

Previously, we demonstrated that OCP3 functions as a negative regulator of JA-dependent disease resistance to necrotrophic fungi [45]. Mutants affected in JA-mediated defenses against necrotrophs often have opposite effects on SA-mediated defenses against biotrophs (reviewed by [2,8]). To study whether *OCP3 *plays a role in the modulation of SA-dependent defenses, we examined the effect of the *ocp3-1 *mutation on disease susceptibility in the context of *Arabidopsis *genotypes that are impaired in their ability to produce, accumulate or perceive SA in response to *Pst *DC3000 infection. For this purpose we generated a series of double mutants, including *ocp3-1 pad4-1*, *ocp3-1 *NahG and *ocp3-1 npr1-1 *and evaluated the growth of *Pst *DC3000. *PAD4 *encodes a lipase-like protein that participates in a positive regulatory loop for increasing SA levels in response to pathogen attack, thereby activating SA-dependent defense responses; the *pad4 *mutant thus shows enhanced disease susceptibility to biotrophic pathogens [50]. Transgenic NahG plants expressing the bacterial *nahG *gene (encoding a SA hydroxylase) are unable to accumulate SA and therefore they are also highly susceptible to biotrophic pathogens [25,51]. Finally, mutant plants affected in the central regulator *NPR1 *are compromised in SA perception and similarly show enhanced disease susceptibility to biotrophic pathogens [52]. Figure 1a shows that *pad4-1*, NahG, and *npr1-1 *plants show enhanced susceptibility to *Pst *DC3000 infection, confirming previous findings [25,50-52]. The *ocp3-1 *plants did not differ from Col-0 plants in the level of disease susceptibility to *Pst *DC3000. In the *pad4-1, npr1-1*, and NahG background, the *ocp3-1 *mutation did not alter the level of susceptibility to *Pst *DC3000. These results indicate that, while *OCP3 *is critical for the regulation of JA-dependent basal defenses against necrotrophs [45], it seems not to affect disease resistance against the biotroph *Pst *DC3000.

**Figure 2:**
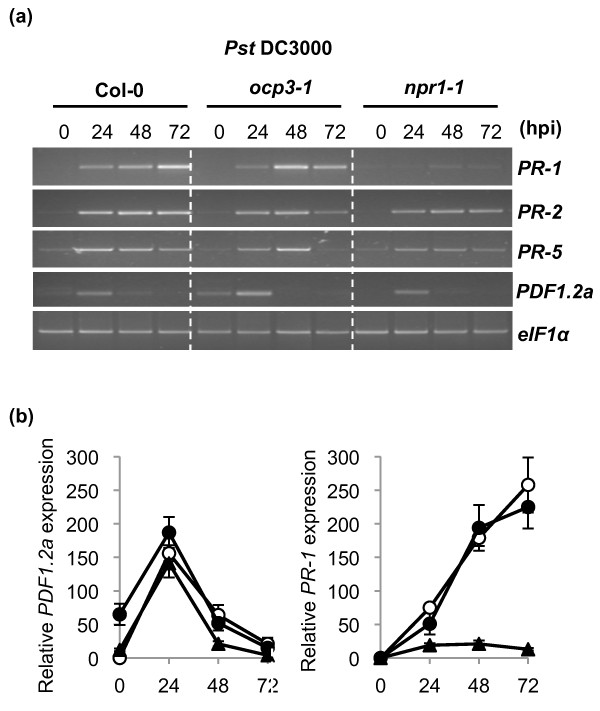
**Comparative expression analysis of SA- and JA-responsive marker genes in Col-0, *ocp3-1 *and *npr1-1 *genetic backgrounds**.** (a)** Time-course gene expression by RT-PCR of some SA- (*PR1, PR2 *or *PR5*) and JA- (*PDF1.2a*) marker genes in response to *Pst *DC3000 infection. Gels were satined with ethidium bromide. **(b)** Time-course gene expression by qRT-PCR of *PR1*and *PDF1.2a *marker genes in response to *Pst *DC3000 infection. Open circles represent expression level in Col-0 plants, filled circles represent expression level in *ocp3-1 *and triangles represent expression level in *npr-1*. The experiments were repeated three times with cDNAs derived from independent biological samples. The housekeeping gene *eIF1α *was used as reference.

Stomata play an active role in limiting bacterial invasion as part of the plant innate immune system [53]. Under natural conditions, *Pst *DC3000 enters host plants, usually the leaves, through these natural openings and wounds, and then spreads and multiplies to high population densities in intercellular spaces [54]. Thus, the infiltration of bacteria with a syringe, as in the experiments shown in Figure 1a, might bypass the first steps of the natural infection process, notably the steps reported to be regulated by ABA [55]. We have recently reported that OCP3 negatively regulates ABA-dependent stomatal closure [49], so the possibility still exists that by infiltrating *Pst *DC3000 directly in the apoplast, as we did in the experiment shown in Figure 1a, we may have overcome a line of disease resistance control associated to the *ocp3-1 *mutation. This thus may mask our interpretation on the results. To study this effect we repeated the experiments and infected *Arabidopsis *plants by spraying *Pst *DC3000 onto the leaf surface and subsequently monitored growth of *Pst *DC3000 (Figure 1b). As can be deduced by comparing the bacterial growth responses shown in Figure 1a, b, both types of inoculation render the same results, thus indicating that OCP3 does not play a role in stomata-mediated defenses. Gene expression analyses in response to *Pst *DC3000 infection, as shown in Figure 2a, revealed that *ocp3-1 *plants show constitutive expression of the JA marker gene *PDF1.2a *which was followed by a further transient induction that peaked at 24 h upon pathogen inoculation. This transient induction also occurs in wild type plants. SA-inducible genes (e.g., *PR-1*, *PR-2 *and *PR-5*), showed no major differences between Col-0 and *ocp3-1 *plants. However, this is in contrast to what is observed in *npr1-1 *plants, which are blocked in *PR-1 *gene expression (Figure 2a). Moreover, activation of *PR-1 *following *Pst *DC3000 infection resulted in the suppression of JA-dependent gene expression (Figure 2a). These experiments were also validated by qRT-PCR (Figure 2b).

### JA- but not SA-induced defenses are compromised in *ocp3-1*

**Figure 3:**
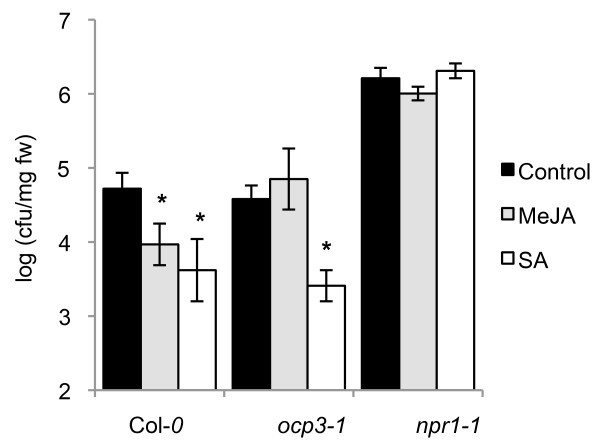
**JA- but not SA-induced disease resistance against *P. syringae *is impaired in the *ocp3-1 *mutant**. Two-week-old plants were treated by dipping with a solution of 50 μM MeJA (light grey), 50 μM SA (white) or MgSO_4 _as control treatment (black). After two days, plants were challenge inoculated as described in Figure 1b. Asterisks indicate significant difference from the control treatment (P < 0.05) using the Student's *t *test. The experiments were repeated a minimum of three times with similar results.

In *Arabidopsis*, exogenous application of SA induces SAR against different types of pathogens (for review, see [24]). Likewise, exogenous application of methyl JA (MeJA) has been shown to induce resistance against *Pst *DC3000 [26,56,57]. Both SA- and JA-mediated induced resistance against *Pst *DC3000 has been shown to require NPR1 [26]. To further examine the possible role of *OCP3 *in the SA- and JA-mediated induced resistance responses, we evaluated the protective effect of exogenous applications of SA and MeJA in *ocp3-1 *plants towards *Pst *DC3000 infection. Wild-type Col-0 and mutant *npr1-1 *and *ocp3-1 *plants were treated with either 0.5 mM SA or 50 μM MeJA two days prior to challenge inoculation with *Pst *DC3000. We observed that SA treatment strongly and significantly reduced bacterial growth by at least 10-fold in wild-type and *ocp3-1 *plants but, as expected, not in the SA-insensitive *npr1-1 *mutant (Figure 3). This confirms that SA triggered an effective disease resistance response against *Pst *DC3000 and that the *ocp3-1 *mutation did not interfere with this process.

Conversely, when bacterial growth *in planta *was measured after treatment with MeJA, *ocp3-1 *plants were clearly compromised in their ability to mount an induced resistance against *Pst *DC3000 (Figure 3). While wild-type plants treated with MeJA showed a significant reduction in bacterial growth of 7,5-fold, this protective effect was not observed in *ocp3-1 *plants. Mutant *npr1-1*, which is compromised in both SA-dependent SAR and JA-dependent ISR, showed enhanced susceptibility to *Pst *DC3000 and was unable to mount an induced resistance when treated with MeJA, confirming previous findings [26]. Together, these results suggest that, while *OCP3 *is not involved in SA-mediated defenses, it is required for mounting JA-dependent induced defenses.

### Compromised JA-induced resistance in *ocp3-1 *is not due to a defect in JA-perception

**Figure 4:**
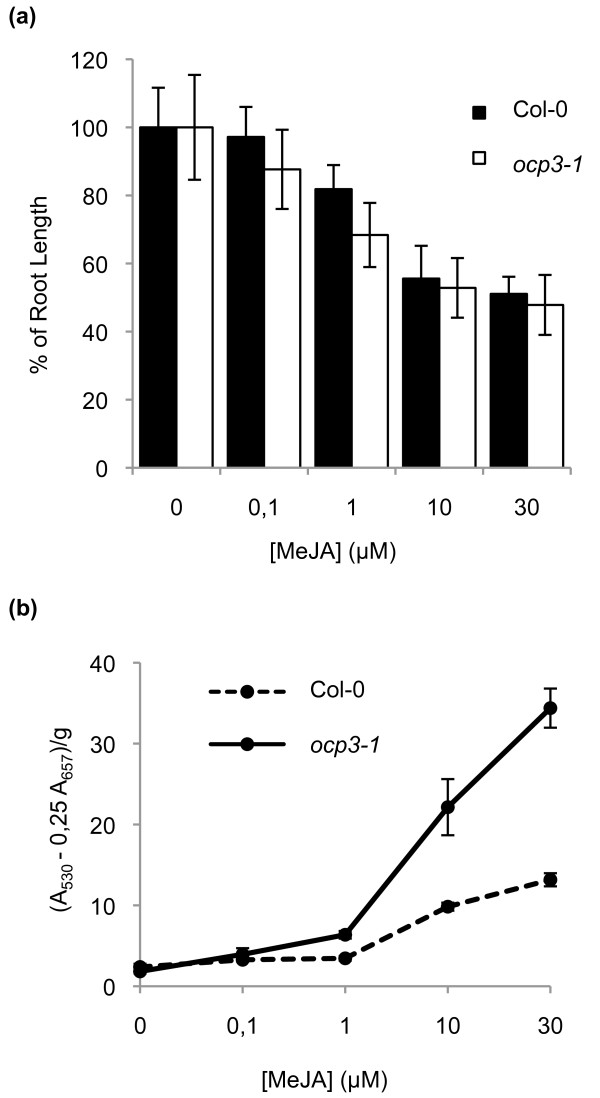
**Mutant *ocp3-1 *is not JA-insensitive**. Effect of JA on Col-0 and *ocp3-1 *plants. Plants were grown in MS plates supplemented with indicated MeJA concentrations during 10 days. Root growth inhibition was represented as the difference to the control (0 μM MeJA) treatment. **(a)** Root growth inhibition. Bars represent the JA-induced rooth-growth inhibition in Col-0 (black bars) and *ocp3-1 *plants (white bars). **(b)** Anthocyanin accumulation. Lines represent the anthocyanin accumulation in the same experiment. Anthocyanin quantification was performed by a spectrophotometric method. Error bars represent standard deviation (n = 5 plates). The experiments were repeated three times with similar results.

Besides its role in plant defense, JA is also implicated in other plant responses, such as the inhibition of root growth [58,59] and the accumulation of anthocyanin (an anti-fungal flavonoid) [60,61]. To further study the responsiveness of *ocp3-1 *plants to JA, we searched for a differential effect that MeJA may have on *ocp3-1 *when compared to Col-0 plants. As shown in Figure 4, the MeJA-induced inhibition of root growth in Col-0 and *ocp3-1 *plants was very similar, indicating that the *ocp3-1 *mutation does not interfere with JA perception. However, upon treatment with MeJA, *ocp3-1 *plants induced and accumulated higher amounts of anthocyanins, suggesting that *ocp3-1 *plants could be altered in a specific defense-related branche of the JA response, without having an effect on development-related processes.

### Mutant *ocp3-1 *is impaired in *P. fluorescens *WCS417r-triggered ISR against *P. syringae *and *H. arabidopsidis*

**Figure 5:**
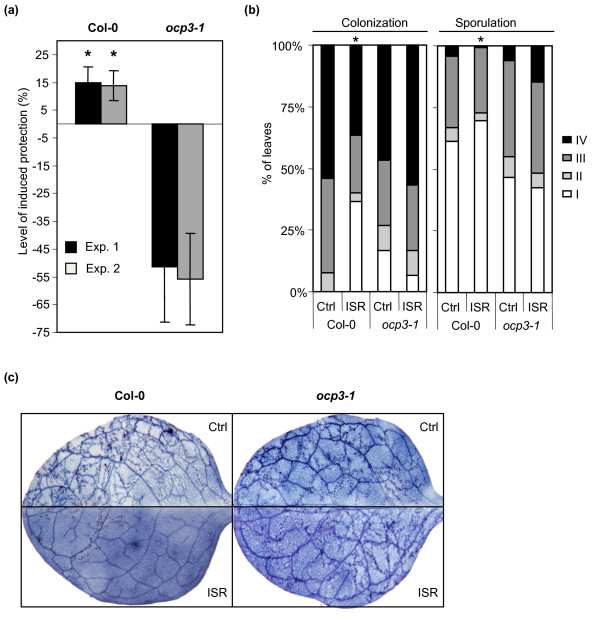
**Mutant *ocp3-1 *plants are not able to mount ISR against *P. syringae *and *H. arabidopsidis***. **(a)** Quantification of *P. fluorescens *WCS417r-mediated ISR against *Pst *DC3000 in *Arabidopsis *Col-0 and *ocp3-1 *plants. The level of induced protection calculated on the basis of the reduction in disease symptoms relative to challenged, non-induced plants. Asterisks indicate statistically significant levels of protection compared to non-induced control plants (Students *t*-test: α = 0.05; n = 20 plants). Data presented are means from two independent experiments. Error bars represents standard errors. **(b)** Quantification of colonization and sporulation of *H. arabidopsidis *infected plants (From class I = no colonization/sporulation to class IV = high colonization/sporulation). Asterisks indicate statistically significant distributions of the disease severity classes compared to noninduced control treatments (Chi-square, P < 0.05; n = 200 leaves). The data presented are from a representative experiment that was repeated with similar results. **(c)** Lactophenol trypan-blue stained control- and *P. fluorescens *WCS417r-treated Col-0 and *ocp3-1 *plants 7 days after inoculation with *H. arabidopsidis*. *Pst *DC3000 inoculation experiments were repeated three times and twice for *H. arabidopsidis *always with a similar out come.

Rhizobacteria-mediated ISR has been studied extensively in *Arabidopsis *using the beneficial rhizobacterial strain *P. fluorescens *WCS417r as the inducing agent and *Pst *DC3000 as the challenging pathogen ([29]for latest review). In Arabidopsis, WCS417r-ISR is an induced JA- and NPR1-dependent defense response that functions independently of SA. To investigate the role of OCP3 in WCS417r-ISR, Col-0 and *ocp3-1 *plants were grown in soil containing either ISR-inducing WCS417r bacteria or MgSO_4 _as a control. Subsequently, plants were inoculated with *Pst *DC3000 and 4 days later the level of induced protection was determined. Figure 5a shows that WCS417r induced a significant level of resistance against *Pst *DC3000 in Col-0 plants. However, treatment of *ocp3-1 *plants with WCS417r resulted in increased susceptibility, rather than in induced protection to *Pst *DC3000.

To corroborate this finding we tested the role of OCP3 in WCS417r-ISR against the oomycete pathogen *H. arabidopsidis*, which has been shown to be sensitive to WCS417r-ISR [35]. To this end, Col-0 and *ocp3-1 *plants were grown in soil with or without WCS417r bacteria and were subsequently inoculated with *H. arabidopsidis. *Figure 5b and 5c shows that the level of colonization and sporulation by the pathogen at 7 days after inoculation was significantly reduced in WCS417r-treated Col-0 plants. However, in mutant *ocp3-1 *this induced resistance was not apparent. *H. arabidopsidis *colonized the leaf tissue of control- and WCS417r-treated *ocp3-1 *plants to the same extent (Figure 5c), indicating that *ocp3-1 *is blocked in its ability to mount ISR.

These results demonstrate that *ocp3-1 *plants are impaired in their ability to mount a proper ISR response against *Pst *DC3000 and *H. arabidopsidis*, suggesting that OCP3 plays a role in the regulation of this JA-dependent induced resistance response.

### Overexpression of an engineered cytosolic NPR1 isoform restores the impaired JA-induced disease resistance in *ocp3-1 *plants

**Figure 6:**
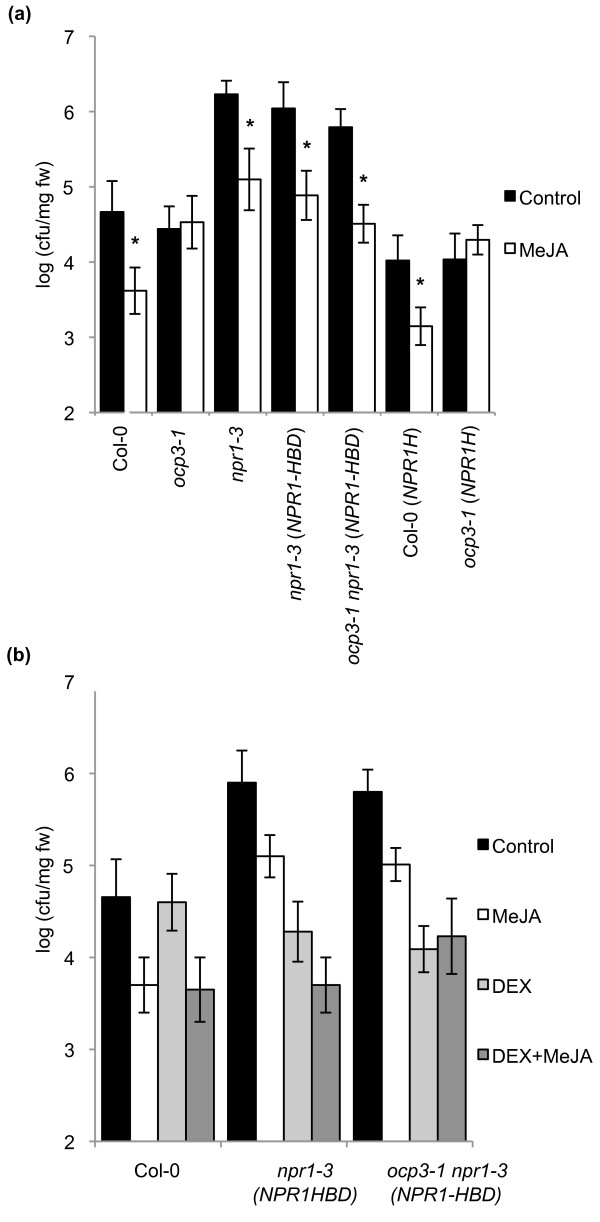
**Overexpression of a cytosolic isoform of NPR1 (NPR1-HBD) restores the impaired JA-induced disease resistance in *ocp3-1 *plants while dexamethasone treatment eliminates this protective effect**. **(a) **Two-week-old plants were treated by dipping with a solution of 10 mM MgSO_4 _(Control) or 50 μM MeJA. After two days, plants were challenge inoculated as described in Figure 3. Asterisks indicate significant difference from the control treatment (P < 0.05) using the Student's *t *test. **(b) **As in (a), plants were treated with 10 mM MgSO_4 _(Control), with 50 μM MeJA, with 5 μM dexamethasone (DEX) or with 50 μM MeJA plus 5 μM dexamethasone (DEX). The experiments were repeated three times with similar results.

In order to study a possible link between NPR1 and OCP3 in the context of JA-dependent defenses, we examined the ability to mount JA-induced resistance of engineered *ocp3-1 *mutant plants that either overexpress the wild-type NPR1 protein (named NPR1-H) or alternatively a fusion of NPR1 with the hormone binding domain of the rat glucocorticoid receptor (named NPR1-HBD) that is impeded in its translocation into the nucleus [18]. As expected, overexpression of *NPR1 *rendered enhanced basal resistance towards *Pst *DC3000 (growth in control-treated plants 0.5 log units lower in Col-0 (NPR1-H) in comparison to Col-0). However, this overexpression had no significant effect on MeJA-induced disease resistance (Figure 6a). Overexpression of *NPR1 *in the *ocp3-1 *mutant background resulted in the same enhanced level of basal disease resistance towards *Pst *DC3000 (in comparison to Col-0), but the MeJA-induced resistance remained blocked in *ocp3-1*(NPR1-H) plants (Figure 6a).

Previously, a cytosolic function of NPR1 was suggested to play a role in the regulation of JA-dependent defense responses [18,38-42]. We took advantage of the available transgenic line, originally generated in a *npr1-3 *background, that overexpress the NPR1 protein as a fusion to the rat glucocorticoid receptor HBD (NPR1-HBD) to genetically perform crosses with *ocp3-1 *plants and generated a homozygous *ocp3-1 npr1-3*(*NPR1-HBD*) line. As expected, and as shown in Figure 6a, overexpression of the cytosolic version of NPR1 (NPR1-HBD) in *npr1*-3 plants had no effect on the basal level of resistance to *Pst *DC3000. This suggests that the NPR1-dependent basal defenses against *Pst *DC3000 require the nuclear function of NPR1. By contrast, the MeJA-induced resistance against *Pst *DC3000 was restored in *npr1-3(NPR1-HBD) *plants (Figure 6a), indicating that cytosolic NPR1 plays a role in regulating JA-induced defenses against *Pst *DC3000 even in the absence of NPR1 in the nuclei. Surprisingly, also in the *ocp3-1 npr1-3 *double mutant background, overexpression of NPR1-HBD restored MeJA-induced resistance against *Pst *DC3000. The observed results suggest that the expression of the cytosolic form of NPR1 (NPR1HBD) in an *ocp3-1 *background recovers the protective effect mediated by treatment with MeJA. Consequently, we further investigate this effect by treating transgenic lines with dexamethasone (DEX) which allows NPR1HBD to enter the nuclei. As shown in Figure 6b, while *npr1-3(NPR1HBD) *plants respond to MeJA treatment in combination with DEX with a further enhancement of resistance towards *Pst DC3000*, this effect on further enhancing resistance is not observed in the case of *ocp3-1 npr1-3(NPR1HBD) *plants. These results point to a model in which the inability of *ocp3-1 *plants to mount an effective JA-induced defense response, including that controlling ISR, could be explained by a defect in controlling the cytosolic function of NPR1 in regulating induced plant defense responses.

## Discussion and Conclusions

OCP3 is an *Arabidopsis *homeodomain transcription factor that negatively regulates a branche of the JA signaling pathway that leads to basal defense against necrotrophic pathogens [45,49].

Most of the mutants reported to be affected in a JA-mediated basal resistance against necrotrophs show opposite effects on the SA-mediated basal resistance against biotrophs. This trade-off is generally explained by the antagonistic action observed between SA and JA on each other signal pathway [3,21,44]. In this respect, however, we observed that *ocp3-1 *plants show enhanced disease resistance to necrotrophic fungi [45] without altering the level of basal resistance to biotrophic pathogens such as *Pst *DC3000 (Figure 1) and *H. arabidopsidis *(Figure 5). Here, we further studied the role of OCP3 in SA- and JA-dependent induced defenses. We have demonstrated that *OCP3 *is not involved in the direct activation of SA-dependent defenses as loss of function of this gene did not interfere with the enhanced susceptibility to *Pst *DC3000 of the SA-related genotypes *pad4-1, npr1-1*, and NahG that are impaired in their ability to produce, accumulate or perceive SA (Figure 1). However, and for NahG plants, we can not disregard the possibility that the accumulation of cathecol may have some negative effects that may neutralize or interfere the outcome of the *ocp3-1 *mutation. In addition, *ocp3-1 *plants showed no defect in the induction of the SA-related marker genes *PR-1, PR-2*, and *PR-5 *upon inoculation with *Pst *DC3000 (Figure 2). OCP3 neither affected the antagonistic effect of SA on JA-dependent gene expression pattern since *PDF1.2a *gene expression in *ocp3-1 *plants remained suppressed upon exogenous SA application [45] or after *Pst *DC3000 infection (Figure 2).

In addition to basal defense, SA and JA signaling pathways are also involved in the regulation of induced disease resistance responses. SA is key signal for pathogen-induced SAR, whereas JA and ET are required for rhizobacteria-mediated ISR. Both types of induced resistance protect the plants against a broad spectrum of pathogens although with a different spectrum of effectiveness [56]. In this study, we provide evidence for a specific function of OCP3 in the regulation of JA- but not SA-induced defenses. This can be deduced from the observation that MeJA is able to induce significant levels of protection against *Pst *DC3000 infection in wild-type plants but not in *ocp3-1 *plants (Figure 3). Moreover, JA-dependent WCS417r-ISR against both *Pst *DC3000 and *H. arabidopsidis *was severely compromised in *ocp3-1 *plants (Figure 5). Together, these results indicate that OCP3 plays an important role in the regulation of JA-dependent induced defense responses to biotrophs. This is in marked contrast with its proposed role as a negative regulator of basal defense against necrotrophic pathogens [56]. These apparent opposite roles, as controlled by the same regulator, demonstrate that OCP3 has distinct functions in basal and induced JA-dependent defenses.

In addition to its role in the activation of SA-dependent basal defenses, NPR1 is a central regulator in the induced defense signaling network that is controlled by the SA and JA/ET interplay. While the nuclear function of NPR1 seems to be required for the regulation of SA-dependent basal defenses and SAR [62,63], a cytosolic function of NPR1 seems to be involved in the modulation of JA-dependent defenses [18,39,40]. Because mutants *ocp3-1 *and *npr1-1 *show similar defects in MeJA-induced disease resistance and rhizobacteria-mediated ISR, we could not study the existence of a possible epistatic relationship with the double *ocp3-1 npr1-1 *mutant. Alternatively, we used transgenic plants overexpressing either the wild-type NPR1 protein (NPR1-H, [64]) or an engineered NPR1 version (NPR1-HBD) that is unable to translocate to the nucleus [18,65]. This approach revealed that although nuclear localization of NPR1 is required for basal resistance against *Pst *DC3000, it is not required for the induction of JA-dependent defenses against *Pst *DC3000. In fact, transgenic *ocp3-1 *plant over-expressing NPR1 remained compromised in mounting an effective JA-induced defenses against *Pst *DC3000 (Figure 6a), whereas overexpression of cytoplasmically-located NPR1-HBD was sufficient to restore the compromised JA-induced defense response against *Pst *DC3000 (Figure 6a). Moreover, while control plants respond to DEX treatment, which in turn releases the cytosilic-retained protein into the nuclei, with a further enhancement of resistance to *Pst *D3000 upon MeJA application (Figure 6b), *ocp3-1 *plants do not show such an enhancement in resistance. These observations reconcile with previous evidences showing a role for cytoplasmatically-located NPR1 in the modulation of JA-dependent induced defense responses [18,38-40,42]. These results point to a model in which OCP3 functions as a modulator of the cytosolic function of NPR1, which in turn may regulate the induction of JA-dependent induced defenses, including ISR. This is in agreement with the observation that only over-expression of cytosolic NPR1 in an *ocp3-1 *background restores MeJA-induced disease resistance against *Pst *DC3000.

Recently, it has been demonstrated that pathogen-triggered redox changes finely regulates NPR1 functions via protein modifications [62,63]. NPR1 is sequestered in the cytoplasm as an oligomer through intermolecular disulfide bonds and is translocated to the nucleus upon SA-mediated monomerization, a process shown to be essential for SA-induced *PR-1 *gene expression. S-nitrosylation of NPR1 by S-nitrosoglutathione (GSNO) facilitates its oligomerization, which maintains protein homeostasis in the cytoplasm upon SA induction. Conversely, the SA-induced NPR1 oligomer-to-monomer reaction is catalyzed by thioredoxins (TRXs). Thus, the regulation of NPR1 functions through the opposing action of GSNO and TRX [63]. According to this mechanism, both cytosolic NPR1 function controlling JA-dependent induced defenses and nuclear NPR1 function controlling SA-dependent defenses must be modulated by pathogen-triggered NO-mediated changes in the redox status of the challenged cell [63].

Our current understanding does not allow us to determine how OCP3 could be modulating the NPR1-mediated JA-dependent activation of defenses. However, it could be the case that OCP3 is regulating specific aspects of the oxidative plant cell status that in turn modulates the cytosolic function of NPR1 required for the activation of JA-dependent induced defenses. In this regard, it has been suggested that OCP3 may be functioning as a specific regulator of the redox homeostasis in plant-pathogen interactions [45]. In fact, *ocp3-1 *mutant plants constitutively express GST1, a glutathione S-transferase implicated in the protection of oxidative stress during several biotic and abiotic plant stresses [66]. In addition, OCP3 could function as a negatively regulator of pathogen-triggered NO accumulation as *ocp3-1 *plants show reduced NO accumulation in response to *Pst *DC3000 infection (Ramírez and Vera, unpublished results). This could be linked to the observation that GSNO, a NO donor, mediates S-nitrosylation of NPR1 to maintain protein homeostasis in the cytoplasm upon SA induction [62]. All these observations reinforce the consideration of the existing link between pathogen-triggered redox changes, as mediated by NO, and the modulation of the NPR1 pool in the cytoplasm. Whether OCP3 may be directly involved in modulating the cytosolic funtion of NPR1, or may be indirectly participating in controlling an exquisite cytosolic environment for NPR1 to exert its cytoslic function still remains unknown. In any case, our finding that OCP3 is pivotal for JA- and NPR1-dependent induced defenses, along with the observation of a degree of genetic epistasis between OCP3 and NPR1, favors the interpretation that the former may be controlling critical functional aspects of the later at least in the cytosol. Understanding how this interplay occurs is our next challenge for the future.

## Methods

### Plant materials and growth

Seeds of mutants and wild-type *Arabidopsis thaliana *were kept at 4°C for 3 days and sown in jiffy7 peat pellets (Clause-Tezier Ibérica, Paterna, Spain) or on a turf substrate mix. Plants were grown in a growth phytochamber with a light intensity of approximately 150-200 μE m^-2^s^-1 ^at 23°C under 10 h light/14 h dark cycles and 60% humidity.

PCR-based detection of *ocp3-1, pad4-1, and NahG *were performed as described [45]. For the *npr1-1 *mutant allele, the primers used were (5'-ATGTCTCGAATGTACATAAGGC-3' and 5'-CTCAGTTTCCTAATAGAGAGG-3'). Genomic DNA was extracted from young leaves of *Arabidopsis *as described [67]. The 581 bp PCR product was digested with *NlaIII *(New England Biolabs) resulting in 263, 204, 98 and 16 bp in wild-type and 302, 263 and 16 bp in *npr1-1*. For the *npr1-3 *mutant allele the primers used were (5'-AGGCCGACTATGTGTAGAAATACTAGTA-3' and 5'-GCAAGTGCAACTAAACAGTGG-3'). The 245 bp PCR product was digested with *RsaI *(New England Biolabs) resulting in 218 and 27 bp in wild-type and 245 bp in *npr1-3*. For the detection of 35S:NPR1 and 35S:NPR1-HBD, the primers used were (5'-AATATCCCGGAGCAATGCAA-3' and 5'-CGGTTGATTTCGATGTGGAAG-3').

In double mutant analysis the same phenotype was observed for, at least, two independent double mutant lines generated.

*ocp3-1 (NPR1-H) *and *ocp3-1 npr1-3 (NPR1-HBD*) lines were generated by crossing *ocp3-1 *plants (in a Col-0 background) with Col-0 (*NPR1-H*) and *npr1-3 (NPR1-HBD*), respectively.

### *Pseudomonas syringae *pv. *tomato *DC3000 bioassays

For preparation of the inoculum, bacteria were streaked out from a -80°C glycerol stock onto a plate of King's medium B supplemented with 100 μg/mL rifampicine and grown for 2 days at 28°C. Bacteria were harvested in 10 mM MgSO_4 _and adjusted to the indicated OD_600 _(In spray inoculations, 0.02% (v/v) Silwet L77 was used as a surfactant). Five-week-old plants were challenge inoculated and three days later, the bacterial growth was measured. Bars represent the logarithm of colony forming units per mg of fresh weight. Error bars represent standard deviation (n = 8)

### Induction treatments

Induction treatments with salicylic acid (SA), methyl jasmonate (MeJA) and 1-aminocyclopropane-1-carboxylate (ACC) were performed 3 days before challenge inoculation by spraying the leaves with a solution containing either SA, MeJA or ACC in 0.02% (v/v) Silwet L77. Control-treated plants were sprayed with a solution containing only 0.02% (v/v) Silwet L77. When indicated, plants were spray-treated with 5 μM dexamethasone (DEX) (Sigma).

### Analysis of JA sensitivity

Seeds of Arabidopsis were surface-sterilized for 2 min in 70% ethanol and 5 min in 5% sodium hypochlorite, washed five times with sterilized water. Subsequently, seeds were distributed evenly on square Petri dishes containing 2.2 g/L MS (Duchefa Biochemie), 5 g/L sucrose, 6 g/L Agargel (Sigma-Aldrich, Steinheim, Germany) (pH 5.7). MeJA (Sigma-Aldrich, Steinheim, Germany) was added to the autoclaved medium from a filltersterilized 1 mM stock solution (containing 0.96% ethanol). Seeds were pre-germinated in the dark for 4 days at 4°C. The effect of MeJA on primary root growth was determined essentially as described by [57]. Plates were incubated vertically in a climate chamber at 22°C with an 8 h day (approximately 200 μE m^-2 ^sec^-1^) and a 16 h night cycle. After 10 days plates were photographed and the primary root length was measured using the free software ImageJ 1.36b (Broken Symmetry software). In each case, 5 plates were measured (30 seedlings/plate).

### Extraction and quantification of anthocyanins

Extraction and quantification of anthocyanins was performed in accordance with the protocols of [68], with minor modifications.10-day-old seedlings grown as described above in MS medium supplemented with the indicated MeJA concentrations were collected and homogenized in one milliliter of acidic methanol (1% [w/v] HCl) (6,7 mL HCl in 250 mL Methanol) was added to 0.3 g of fresh seedling tissue. Samples were incubated for 18 h at 21°C under moderate shaking (95 rpm). After centrifugation (21,500 *g*, room temperature, 3 min), 0.4 mL of the supernatant was added to 0.6 mL of acidic methanol. Absorption of the extracts at wavelengths of 530 and 657 nm was determined photometrically (Biophotometer, Eppendorf). Quantitation of anthocyanins was performed using the following equation: *Q *(anthocyanins) = (*A*_530 _- 0.25 *A*_657_) × *M*^-1^, where *Q *(anthocyanins) is the concentration of anthocyanins, *A*_530 _and *A*_657 _are the absorptions at the wavelengths indicated, and *M *is the fresh weight (in grams) of the plant tissue used for extraction. The numbers of samples used for the measurements are indicated in each figure. Error bars indicate the SD of the average anthocyanin contents.

### RNA isolation, RT-PCR and qRT-PCR analysis

RNA was isolated with Trizol (Invitrogen). For RT-PCR, RevertAid M-MuLV Reverse Transcriptase (Fermentas) was used according to the manufacturer's instructions. The resulting single stranded cDNA was then used as template in semi-quantitative PCR (RT-PCR). RT-PCRs were carried out with gene specific primers designed using the Primer Express 2.0 software (Applied Biosystems) (*PR-1 *(*AT2G14610*): 5'-ATGAATTTTACTGGCTATTC-3' and 5'-AACCCACATGTTCACGGCGGA-3', *PR-2 *(AT3G57260): 5'-GCTTCCTTCTTCAACCCCACA-3' and 5'-CTGAACCTTCCTTGAGACGGA-3', *PR-5 *(AT1G75040): 5'-CTCTTCCTCGTGTTCATCACA-3' and 5'-CATCTACGAGGCTCACATCGT-3', *PDF1.2a *(AT5G4442): 5'-ATGGCTAAGTTTGCTTCCAT-3' and 5'-ACATGGGACGTAACAGATAC-3', *eIF1α *(AT5G60390): 5'-GCACAGTCATTGATGCCCCA-3' 5'-CCTCAAGAAGAGTTGGTCCCT-3'. qRT-PCRs were carried out with gene specific primers designed using the Primer Express 2.0 software (Applied Biosystems): PR-1: 5'-AAGGGTTCACAACCAGGCAC-3' and 5'-CACTGCATGGGACCTACGC-3'; PDF1.2a: 5'-CTTGTTCTCTTTGCTGCTTTC-3' and 5'-CATGTTTGGCTCCTTCAAG-3'. qRT-PCRs were performed using the SybrGreen PCR Master Mix (Applied Biosystems) in a ABI PRISM 7000 sequence detector. Cts were obtained using the 7000 System SDS Software Core Application Version 1.2.3 (Applied Biosystems) and the data was transformed with the formula 2^^(40-Ct)^. qRT-PCR and RT-PCR analyses were performed at least three times using sets of cDNA samples from independent experiments.

### *P. fluorescens *WCS417r-triggered ISR bioassays

In these experiments, *Arabidopsis thaliana *accessions Columbia (Col-0) and the mutant *ocp3-1 *were sown in quartz sand. Two-week-old seedlings were transferred to 60-mL pots, containing a sand/potting soil mixture that had been autoclaved twice for 20 min. Plants were further cultivated, as described [69]. For treatment of the roots with ISR-inducing rhizobacteria, *Pseudomonas fluorescens *WCS417r was grown on King's medium B agar plates [70] for 24 h at 28°C. Bacterial cells were collected by centrifugation and resuspended in 10 mM MgSO_4 _to a final density of 10^9 ^colony-forming units (cfu) per mL. ISR was induced by transplanting 2-week-old *Arabidopsis *seedlings to soil supplemented with a suspension of WCS417r bacteria to a final density of 5 × 10^7 ^cfu/g as described [69].

### *H. arabidopsidis *bioassays

*H. arabidopsidis *WACO9 sporangia were obtained by washing sporulating Col-0 leaves in 10 mM MgSO_4_, collected by centrifugation, and resuspended in 10 mM MgSO_4 _to a final density of 5 × 10^4 ^sporangia per mL as described [54]. Three-week-old seedlings were challenge inoculated with *H. arabidopsidis *by spraying with 10 mM MgSO_4 _containing 5 × 10^4 ^conidiospores per mL. Inoculated plants were maintained at 17°C and 100% relative humidity. Disease symptoms were scored for about 200 leaves per treatment at 7 days after challenge. Disease was monitored by assessing the rate of colonization and sporulation as described [56]. For determining leaf colonization, infected leaves were stained with lactophenol trypan-blue and examined microscopically at 7 days after inoculation, as described by [71] and scored on each leaf in the following classes: I, no colonization; II, low tissue colonization (<25% of leaf area colonized); III, medium tissue colonization (25-50% of leaf area colonized); IV, high tissue colonization (>50% of leaf area colonized). Sporulation was expressed as intensity of pathogen sporulation on each leaf: I, no sporulation; II, <50% of the leaf area covered by sporangiophores; III, >50% of the leaf area covered by sporangiophores; and IV, heavily covered with sporangiophores, with additional chlorosis and leaf collapse.

### Lactophenol trypan-blue staining

Leaves were plunged in lactophenol trypan blue (30 mL ethanol, 10 mL glycerol, 10 mL lactic acid, 10 mg trypan blue and 10 mL distilled water) and boiled at 95°C for 2-3 min and then incubated at room temperature for 1 h. Samples were transferred into chloral hydrate solution (2.5 g mL^-1^) and boiled about 20 min. After several exchanges of hydrate chloral solution, samples were equilibrated in 50% (w/v) glycerol and observed using a light microscopy.

## Authors' contributions

VR carried out the molecular and genetic studies, the *P. syringae *infection assays, participated in the design of the study. SVE carried out the *P. fluorescens *WCS417r-triggered ISR bioassays. AC participated in the genetic studies and in the JA assays. CMJP participated in the design of the study and critically revised the manuscript. PV conceived of the study, participated in its design and coordination of experiments. VR and PV wrote the manuscript. All authors read and approved the final manuscript.
